# Jost Steinhäuser: PraxisSkills Allgemeinmedizin

**DOI:** 10.3205/zma001091

**Published:** 2017-05-15

**Authors:** Matthias Angstwurm

**Affiliations:** 1 Klinikum der LMU München, Medizinische Klinik und Poliklinik IV, München, Deutschland

## Recension

Very tightly focused on clinical practice, this book covers in detail basic skills that may be required of general practitioners, particularly if they do not practice in an urban center, but rather in a rural area where physicians must rely more heavily on themselves. The typical activities of a general practitioner are described ranging from taking blood and delivering babies to various puncturing and suturing techniques, from minor surgeries to emergency medicine.

Worthy of note is that most chapters have been written by multiple authors, usually a team consisting of a general practitioner and a medical specialist resulting in the excellent quality of the descriptions and illustrations.

This easy to handle book describes how procedures are carried out step by step. These descriptions are often augmented by a discussion of theoretical aspects in an attempt to expand the potential readership of students and professionals in medical occupations. In part, the demand for pre-existing knowledge is very low, as demonstrated by the description of how to take a blood sample. On the other hand, relatively complex procedures are included, such as the removal of a lipoma, something that is more likely to fall within the scope of a specialist or advanced post-licensure training. Gynecological procedures and maneuvers are presented in depth and in the manner of a textbook.

As a result, the book's target audience is not clearly identifiable. Since the basic skills also include changing bandages, taping and suturing techniques, primarily fifth-year medical students or those preparing to practice general medicine will profit from this book. However, to learn practical skills, students must be guided through procedures by a mentor, and this kind of guidance cannot come from a book or video. Hence, this book cannot act as a substitute for teaching practical skills.

All of the chapters follow the same very clear organization and provide information on indications, courses of action, contraindications and complications. In addition, passages from the literature and links to video material are integrated into the material so that the skills can be illustrated even more closely as they are practiced and can be learned in even more detail.

These links to URLs, however, can only be used if the reader has registered with the Schattauer Verlag. Some of the videos require registration with other publishers or services, such as mediaservice.bbraun.de. Consequently, the use of what are certainly valuable resources will be clearly reduced. A much simpler method to provide teaching materials without requiring the disclosure of personal information to the publishers would be an internet platform created, for instance, by the book's publisher, the Schattauer Verlag, through which all the additional materials and literature can be accessed with a code that is provided along with the purchase of the book.

## Competing interests

The author declares that he has no competing interests.

